# Rethinking genomics of facioscapulohumeral muscular dystrophy in the telomere-to-telomere era: pitfalls in the hidden landscape of D4Z4 repeats

**DOI:** 10.1038/s41431-025-02000-x

**Published:** 2026-01-14

**Authors:** Valentina Salsi, Francesca Losi, Sara Pini, Matteo Chiara, Rossella Tupler

**Affiliations:** 1 Department of Biomedical, Metabolic and Neural Sciences, University of Modena and Reggio Emilia, Modena, Italy; 2 Department of Biosciences, University of Milan, Milan, Italy

**Keywords:** Neuromuscular disease, Genome assembly algorithms

## Abstract

Facioscapulohumeral muscular dystrophy (FSHD) is genetically associated with reduction of the D4Z4 macrosatellite array at 4q35 on a permissive 4qA haplotype, a configuration that enables the stable expression of the DUX4 transcription factor. Current diagnostic and mechanistic models, however, rely heavily on the incomplete GRCh38/hg38 reference and assume that D4Z4 repeats are predominantly confined to 4q35 and 10q26 loci. Here we present a systematic re-analysis of the configuration of D4Z4-like repeats in the human genome using the Telomere-to-Telomere human genome assembly (T2T-CHM13 v2.0/hs1) and complementary experimental validation. Using the terminal 4q35 repeat as a query, we annotated the full repertoire of D4Z4-like loci across the genome and characterized their structural completeness, flanking sequences, and coding potential. This survey uncovered clusters and isolated monomers on at least ten additional chromosomes, several of which harbor intact DUX4 open reading frames or polyadenylation signals. In silico PCR and assays on monochromosomal hybrid cell lines demonstrate that primer sets widely employed for DUX4 or DBE-T detection amplify multiple loci beyond 4q/10q. Together, these findings demonstrate that many signals historically attributed to the pathogenic 4q locus may in fact arise from paralogous arrays. Our study establishes the necessity of locus-resolved, repeat-aware approaches, combining long-read sequencing, methylation-aware profiling, and isoform-resolved transcriptomics, for accurate diagnostics and to define the molecular basis of FSHD.

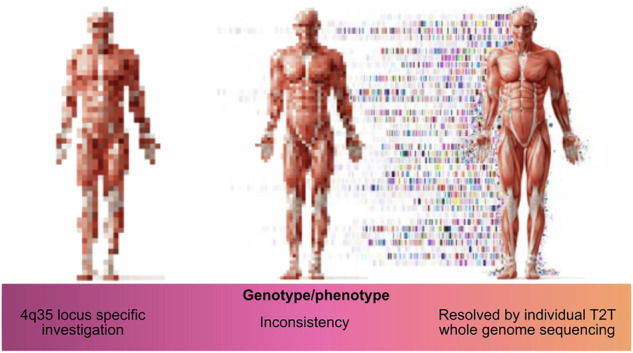

## Introduction

Facioscapulohumeral muscular dystrophy (FSHD) is a genetically and epigenetically complex muscle disorder associated with reduction of the D4Z4 macrosatellite array at the subtelomeric region of chromosome 4q35 [1, 2]. Disease onset is linked to loss of D4Z4 repeats in combination with a permissive haplotype background, termed 4qA/PAS, which enables inappropriate expression of the DUX4 transcription factor [3]. Two main subtypes are recognized, FSHD1 and FSHD2, both of which converge on the aberrant activation of DUX4 [4, 5]. In FSHD1, pathogenicity is associated with reduction of the D4Z4 repeat array, which normally comprises ~100 units but is reduced to fewer than eight in affected individuals. This genetic lesion must occur on a permissive qA-PAS haplotype, defined by the qA sequence, the pLAM region, and a single nucleotide polymorphism (SNP; ATTAAA) that provides a polyadenylation signal for DUX4 transcripts, thereby allowing their stabilization and accumulation. In FSHD2, by contrast, derepression of DUX4 is not due to array contraction but rather to heterozygous mutations in chromatin regulators such as SMCHD1 [6–9], DNMT3B [10], or LRIF1 [11], which act in trans upon a permissive qA-PAS allele, again enabling DUX4 expression [9, 12, 13].

Over the past two decades, both diagnostic criteria and mechanistic models have largely focused on the 4q35 locus as the site of D4Z4 reduction and DUX4 transcription [3, 14]. However, although the existence of sequences homologous to D4Z4 at many other chromosomal locations has been known for a long time [15, 16], these remained largely unexplored primarily because previous genome assemblies excluded many subtelomeric and pericentromeric regions rich in repetitive elements. As a result, FSHD research has historically operated within a locus-centered framework that emphasized 4q35 (and to a lesser extent another almost identical D4Z4 array at 10q26). In contrast, other D4Z4-like repeats had been either undetected or considered to be biologically irrelevant [1, 3, 15].

In 2022, the release of the fully resolved Telomere-to-Telomere human genome reference (T2T-CHM13 CHM13v2.0/hs1) [17], hereafter T2T-CHM13, along with the emergence of pangenomic frameworks [18], allowed the re-evaluation of the D4Z4 landscape across the human genome, including interindividual variability [19]. These resources revealed that D4Z4-like repeats are numerous, structurally diverse, and transcriptionally active, often located in unannotated or structurally variable domains [20]. These discoveries raise the question of whether many diagnostic and research strategies may have inadvertently detected or amplified off-target signals from non-4q/10q loci, with significant implications for data interpretation.

The landscape of repeat-linked transcription at 4q35 is even broader than DUX4 itself. Several studies have demonstrated that D4Z4 units are not transcriptionally silent but are actively transcribed in multiple contexts [21]. Snider et al. (2009) [22] documented bidirectional RNA transcripts extending across several D4Z4 units and mapped the transcription start site of the DUX4-containing transcript to a region immediately upstream of the DUX4 ORF. Block et al. (2012) [23] showed that a region distal to the DUX4 retrogene initiates antisense transcription, which is predominant and dynamically regulated during myogenic differentiation and in FSHD myotubes. Consistent with these findings, Tawil et al. (2014) [24] concluded that each D4Z4 repeat can originate transcripts either near the promoter region of the DUX4 ORF or from more distal elements, extending in both directions across multiple repeats. Together, these studies established that transcription from D4Z4 is pervasive and generates not only full-length DUX4 but also alternative isoforms, small RNA fragments, and even non-canonical protein products.

Moreover, transcription at 4q35 extends beyond DUX4. The long non-coding RNA DBE-T [25], proximal to the D4Z4 array, has been reported to encompass the first chr4q35 D4Z4 repeat and to facilitate local chromatin derepression. More recently, the long non-coding RNA FRG2A, adjacent to the D4Z4 array, was shown to be strongly upregulated in FSHD [26–28]. This places FRG2A in the same category of repeat-linked transcripts as DBE-T and DUX4 itself, exemplifying how derepression at 4q35 can unleash a wider RNA program with architectural and translational consequences.

These observations converge on a central point: many of the tools traditionally used to study FSHD, PCR primers, hybrid cell lines, SNP-based attribution strategies, were developed under the assumption that D4Z4 repeats were restricted to 4q/10q. The completeness of T2T-CHM13 now reveals that this assumption is untenable. Signals previously attributed exclusively to the 4q35 pathogenic locus may in fact represent contributions from multiple paralogous sites. Understanding the biology and pathogenic potential of these repeat elements requires locus-resolved, repeat-aware approaches, including long-read sequencing, isoform-resolved transcriptomics, and methylation-aware assays. Only with these tools can the field disentangle genuine 4q-driven pathogenic events from the broader transcriptional activity of D4Z4-like repeats. In this study, we systematically reassess the genomic distribution, structural organization, and transcriptional competence of D4Z4-like repeats using the T2T-CHM13 assembly in combination with experimental validation on monochromosomal hybrids. We further evaluate the specificity of commonly used primer sets for DUX4 and DBE-T detection, and test the extent to which non-canonical repeats contribute to the pool of D4Z4-derived transcripts.

## Materials and methods

### Annotation of D4Z4-like elements

The complete sequence of a reference D4Z4 repeat (terminal 4q35 repeat) was obtained from the hg38 assembly in UCSC genome browser (https://genome.ucsc.edu/). D4Z4-like elements and functional elements of the D4Z4 array were annotated in the T2T assembly (T2T-CHM13v1.1—Genome—Assembly—NCBI) by sequence similarity searches based on the BLAST program [29] with default parameters. Results of BLAST sequences similarity searches were stored in simple tabular format and processed by custom Perl scripts. A similarity threshold of 85% and an alignment length threshold of 400 or more aligned residues were used to define D4Z4-like elements. D4Z4 arrays were annotated for the presence/absence of the DBE ( > 20 bp), the presence/absence and sequence identity of *DUX4* Exon 1 ( > 1200 bp), and the presence/absence of the pLAM sequence (including *DUX4* Exons 2 and 3 and the integrity of the AUUAAA polyadenylation signal). Manual inspection of the specific flanking was also performed based on the T2T sequence reported in the UCSC Genome Browser (https://genome.ucsc.edu/).

Structurally complete repeats (containing the 5′ promoter region, intact *DUX4* ORF, Exon 2, and Exon 3) but lacking a functional polyA signal, as well as repeats carrying a polyA signal but missing a complete pLAM cassette, were further characterized by predicting the encoded protein through in silico translation using Expasy translation tool (https://web.expasy.org/translate/). The predicted proteins were aligned to DUX4FL using Clustal Omega Multiple Sequence Alignment (MSA) (https://www.ebi.ac.uk/jdispatcher/msa/clustalo?stype=protein), and sequence identity was calculated. Finally, Mulatalin (http://multalin.toulouse.inra.fr/multalin/) was used to generate multiple sequence alignments and graphical multalin plots comparing the predicted proteins with DUX4FL, DUX4-short (DUXsh), and DUX4c.

### CHO hybrid

Human chromosome hybrids (CHO/Hyb) were obtained from the Coriell Institute for Medical Research (https://www.coriell.org/1/NIGMS/Collections/Somatic-Cell-Hybrids) and cultured according to the provider’s instructions.

### DNA extraction, semi-quantitative PCR, and real-time PCR

Genomic DNA was extracted from CHO-hybrid cells using the DNeasy® Blood and Tissue Kit (Qiagen, cat. #69504), according to the manufacturer’s instructions. Primer pairs listed in Supplementary Table 1 were used to amplify genomic fragments. For targets generating amplicons larger than 200 bp, semi-quantitative PCR was performed with 50 ng of genomic DNA using GoTaq® G2 DNA Polymerase (Promega, cat. #M7841) under standard cycling conditions, with annealing temperatures optimized around 60°C. For targets producing amplicons shorter than 200 bp, real-time PCR (RT-PCR) was carried out with 50 ng of genomic DNA using the iTaq™ Universal SYBR® Green Supermix (Bio-Rad, cat. #1725120) on a CFX Connect™ Real-Time PCR Detection System (Bio-Rad).

## Results

### The D4Z4 repeat family: beyond 4q and 10q

**Fig. 1:**
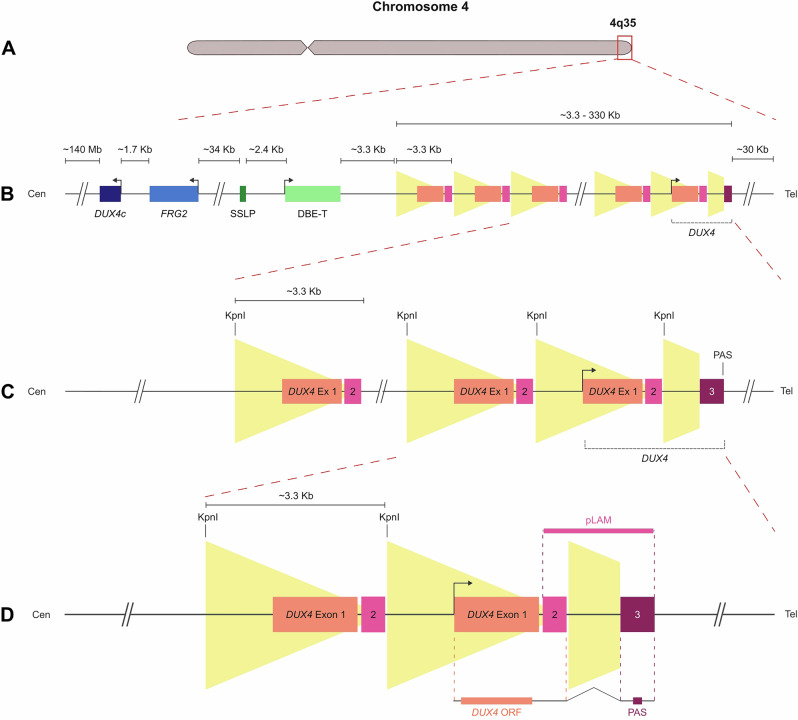
D4Z4 macrosatellite at chromosome 4q35. **A** Ideogram of chromosome 4. The red rectangle indicates the 4q35 region. **B** Enlargement of the 4q35 region (from A) showing the composition of the D4Z4 macrosatellite. **C**, **D** Enlargement of the last four and three D4Z4 repeats. Each canonical D4Z4 repeat is approximately 3.3 kilobases in length, flanked by KpnI restriction sites, and contains exon 1 (orange box), corresponding to the open reading frame (ORF) for DUX4, and exon 2 (pink box). Notably, only the most telomeric (last) repeat in the array is followed by the 3’ UTR-pLAM region (**D**), which contains complete exons 2 and 3 as well as the canonical polyadenylation signal (PAS). This configuration is essential for the generation of stable, disease-relevant DUX4 full-length (DUX4FL).

The D4Z4 macrosatellite consists of tandemly arrayed ∼3.3 kb units, each containing an open reading frame (ORF) encoding the double homeobox transcription factor DUX4 (Fig. 1). At chromosome 4q35, contraction of this array together with a permissive downstream pLAM cassette provides the necessary polyadenylation signal (PAS) for the stabilization of full-length DUX4 (DUX4FL) transcripts, which are considered the canonical pathogenic trigger in facioscapulohumeral muscular dystrophy (FSHD). A highly similar array at 10q26 shares nearly complete sequence identity but is regarded as non-permissive of stable *DUX4* expression, as it lacks the regulatory context required for transcript stabilization. Comparison of the terminal *pLAM* regions reveals that neither 4q35 nor 10q26 harbor a canonical polyadenylation signal (AATAAA*)*. The 4qA haplotype contains a non-canonical ATTAAA motif that can sustain polyadenylation with reduced efficiency, whereas the 10q26 region carries further-degenerated PAS variants (AUCAAA or AATACA) that are largely non-functional. This gradient of PAS efficiency likely contributes to the low abundance and stochastic detection of *DUX4* transcripts, even in permissive genetic backgrounds.

This binary view (4q as pathogenic, 10q as not implicated) has dominated the field for decades. However, the release of the complete T2T-CHM13 genome reveals that D4Z4-like repeats are not confined to 4q/10q but are distributed across multiple chromosomes, including 1, 3, 9, 13, 14, 15, 18, 21, 22, and Y. This expanded landscape raises a critical question: to what extent can signals attributed to 4q truly be considered locus-specific?

**Fig. 2:**
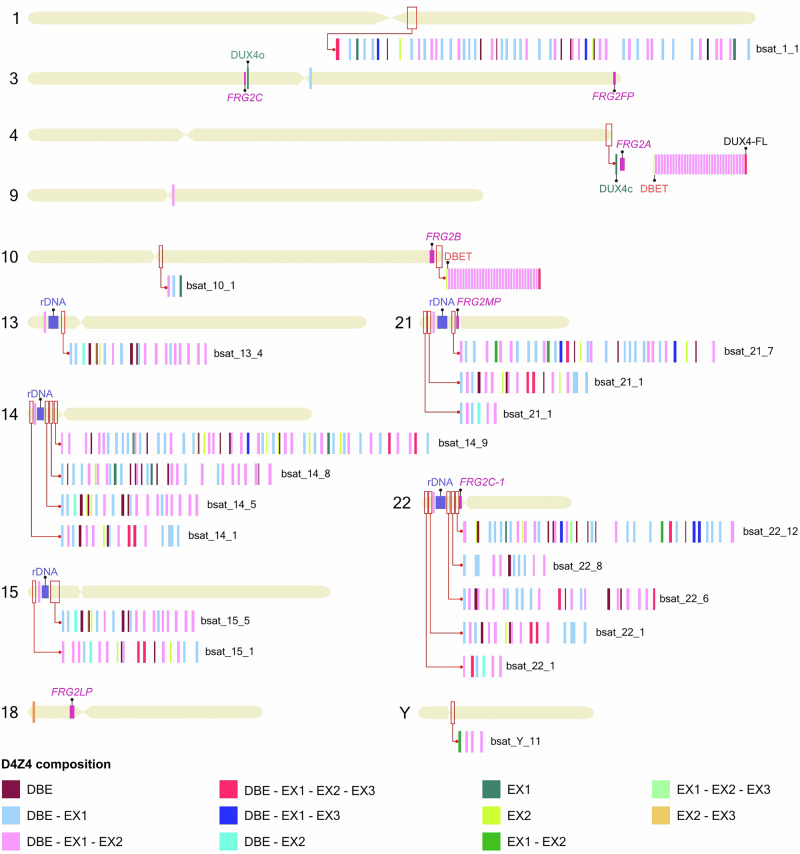
Composition of D4Z4-like sequences and FRG2 family members across the human genome. Chromosome ideograms showing D4Z4-like loci (1, 3, 4, 9, 10, 13, 14, 15, 18, 21, 22, Y). Red rectangles mark the enlarged regions. Stretches of colored boxes represent the composition of D4Z4-like sequences (see legend). Flanking β-satellite sequences (bsat) are shown. DUX4FL (chr4) is indicated. DUX4L9/DUXc (chr4) and DUX4L26/DUXo (chr3) are shown in teal. *FRG2* loci (chr3, 4, 10, 18, 21, 22) are shown in pink. rDNA arrays on acrocentric short arms are shown in blue.

We performed a systematic analysis of D4Z4-like sequences in the T2T-CHM13 assembly, using the terminal 4q35 repeat as a reference and annotating the protein domains and functional elements. The results are summarized in Table 1 and visually represented in Fig. 2, which provides the first integrated genomic map of D4Z4-like arrays together with FRG2 paralogs and rDNA clusters. This overview underscores that these repeats are not isolated but embedded in a broader network of subtelomeric and pericentromeric domains, with potential implications for both transcriptional activity and nuclear organization. Notably, in several chromosomal contexts (e.g., 3q, 4q, 10q, 18q, 21q, 22q), D4Z4-like repeats occur in close proximity to FRG2 paralogs, forming recurrent D4Z4–FRG2 blocks. This repeated association suggests that these elements were duplicated and propagated as modular units during genome evolution, raising the possibility that their transcriptional and epigenetic regulation may also be functionally linked. With this shared ancestry, analysis of restriction-site polymorphisms revealed that *XapI* motifs predominate in the 4q array and *BlnI* in the 10q array, whereas many D4Z4-like repeats on other chromosomes contain recognition sequences for both enzymes. In particular, BlnI sites were predominantly associated with the chr10 repeat array (*n* = 33), whereas XapI sites characterized the chr4 array (*n* = 33). Most repeats located on other chromosomes also contained an XapI site (*n *= 346), while a substantial fraction carried both restriction sites (*n* = 109), including the terminal (telomeric) repeats at both chr4 and chr10. Most likely this pattern reflects local sequence polymorphisms within individual D4Z4 units rather than the presence of mixed or hybrid arrays, supporting the view that the *BlnI/XapI* variants arose secondarily within a common ancestral repeat family [30, 31].

**Table 1:** Genomic and functional annotation of D4Z4-like loci.

Chrom. location	N. of D4Z4-like Units	Flanking seq.	Completeness KpnI/KpnI with promoter	DBE >20 bp	Exon1 (DUX4 ORF) >1200 bp		pLAM		
					Number	Identity %	Exon2	Exon3	PolyA
Chr4 q35	DUX4L9-201 (DUXc)	FRG2_201	1 Partial	0/1	1/1	95.2	1/1	0/1	0/1
Chr4 q35	34	D4Z4 subtel	33 Complete^*^ 1 Partial 1^st^ rep	33/33 0/1	33/33 0/1	99.7-100 n.d.	33/33 1/1	1/33 last rep 0/1	**1/33** 0/1
Chr10 q26	34	D4Z4-subtel	33 Complete^*^ 1 Partial 1^st^ rep	33/33 0/1	33/33 0/1	98.6-99.2 n.d.	33/33 1/1	1/33 last rep 0/1	0/34 0/1
Chr10 q11	3 including DUXL16 & 18	bsat_10_1	3 Partial	2/3	3/3	88.4-90.5	0/3	0/3	0/3
Chr1 q12	57	bsat_1_1	1 Complete^*^ 56 Partial	1/1 34/56	1/1 31/56	89.9 85.9-90.4	1/1 21 /56	1/1 2/56	0/1 0/56
Chr13 p11	26	bsat_13_4	1 Complete^*^ 25 Partial	1/1 20/25	1/1 15/25	89.6 87.8–90.7	1/1 13/25	0/1 0/25	0/1 0/25
Chr13 p13	2	bsat_13_4	2 Partial	1/2	1/2	90.4	1/2	0/2	0/2
Chr14 p13	17	bsat_14_1	17 Partial	16/17	14/17	87.9–90.5	8/17	2/17	0/17
Chr14 p13	2	bsat_14_2	2 Partial	1/2	1/2	90.6	1/2	0/2	0/2
Chr14 p11	26	bsat_14_5	1 Complete^*^ 25 Partial	1/1 19/25	1/1 14/25	89.7 85.9–90.4	1/1 12/25	0/1 0/25	0/1 0/25
Chr14 p11	36	bsat_14_8	2 Complete^*^ 34 Partial	2/2 26/34	2/2 21/34	93.5–96.7 87.4–90.5	2/2 11/34	0/2 0/36	0/2 3/34^**^
Chr14 p11	56	bsat_14_9	56 Partial	46/56	42/56	86.2–90.1	28/56	3/56	1/56^**^
Chr15 p13	19	bsat_15_1	19 Partial	16/19	12/19	86.1–90.2	13/19	2/19	0/19
Chr15 p13	1	bsat_15_2	1 Partial	1/1	1/1	90.7	0/1	0/1	0/1
Chr15 p11	25	bsat_15_5	1 Complete^*^ 24 Partial	1/1 19/24	1/1 14/24	89.5 86–90.9	1/1 12/24	0/1 0/24	0/1 0/24
Chr18 p11	1	Intron-subtel	1 Partial	0/1	0/1	n.d.	1/1	1/1	0/1
Chr21 p13	26	bsat_21_1	26 Partial	23/26	19/26	86.6–90.7	15/26	2/26	0/26
Chr21 p13	2	bsat_21_2	2 Partial	1/2	1/2	90.4	1/2	0/2	0/2
Chr21 p11	38	bsat_21_7	38 Partial	29/38	28/38	86.4–89.8	15/38	3/38	0/38
Chr22 p13	23	bsat_22_1	23 Partial	22/23	19/23	86.4–90.6	14/23	3/23	1/23^**^
Chr22 p13	2	bsat_22_2	2 Partial	1/1	1/1	90.7	1/2	0/2	0/2
Chr22 p11	28	bsat_22_6	28 Partial	25/28	20/28	87.3–91.3	14/28	2/28	2/28^**^
Chr22 p11	11	bsat_22_8	11 Partial	11/11	10/11	87.4–90.8	4/11	0/11	0/11
Chr22 p11	43	bsat_22_12	2 Complete^*^ 41 Partial	2/2 30/41	2/2 26/41	89.5–90.4 88–90.7	2/2 12/41	0/2 5/41	0/2 1/41^**^
Chr3 q11	2	bsat_3_1	2 Partial	1/2	1/2	88.3–90.6	0/2	0/2	0/2
Chr3 p12	DUX4L26-201 (DUXo)	FRG2C_201	1 Partial	0/1	1/1	88.8	0/1	0/1	0/1
Chr9 q12	1	bsat_9_5	1 Partial	1/1	1/1	87.9	0/1	0/1	0/1
ChrY q11	4 DUXL16,17,18 &19	bsat_Y_11	4 Partial	3/4	4/4	89.1–92	4/4	0/4	0/4

(i) Chromosomal location and cytoband;(ii) Copy number and organization (tandem arrays vs isolated monomers);(iii) Flanking sequence context relevant to transcriptional activity and chromatin organization;(iv) Completeness of the repeat unit, defined as the canonical 3.3 kb KpnI–KpnI D4Z4 monomer containing the putative DUX4 promoter (truncated/degenerate elements classified as incomplete);(v) Presence of the D4Z4 Binding Element (DBE; as defined in Methods); (vi) Presence of an Exon 1–containing DUX4 ORF and its sequence identity to the 4q35 last-repeat sequence;(vii) Presence of a pLAM-like downstream cassette (DUX4 exon 2, exon 3, and canonical polyadenylation signal), which together determine the potential to generate full-length DUX4 transcripts.

^*^Complete but lacking the PolyA signal.

^**^Carrying a putative PolyA signal but lacking Exon 3 or Exon 2 and 3.

Our survey reveals heterogeneity in the modular composition of these loci. Some repeats are structurally complete, carrying both the 5′ promoter region and an intact ORF, Exon2, and Exon3, but lack a functional polyA signal. Others harbor a polyA signal but lack a complete pLAM cassette (e.g., missing Exon 2 and Exon 3, or just Exon 3). Within this subset, in addition to the well-characterized chromosome 10 locus, we identified five additional loci with the potential to encode DUX4-related proteins.

Their features, amino acid sequences, and alignments to DUX4FL, DUX4sh, and DUX4c reference sequences are presented in Supplementary Fig. 1. Briefly:

**Chromosome 10 locus:** nearly indistinguishable from 4q35 (99.5% identity), representing the well-known paralogous array at 10q26.

**Chromosome 1 locus:** encodes a putative full-length protein (423 aa) with 80.2% identity to DUX4FL. This sequence contains both homeobox domains and the C-terminal transactivation domain, and could potentially be expressed if a functional polyadenylation signal were provided by local sequence variation. Notably, this paralog shows high similarity to DUX4c (94.65%) but, in contrast to DUX4c, it retains the C-terminal TAD.

**Chromosome 14 loci**:

- *Locus 1* encodes a truncated protein containing only the first homeobox domain, followed by a polyadenylation signal, with 74.9% identity to DUX4FL.
- *Locus 2* encodes a protein of 379 aa with 79.95% identity to DUX4FL, retaining both homeobox domains and the transactivation domain, and also carrying a polyadenylation signal.

**Chromosome 22 loci:**

- *Locus 1* encodes a truncated protein with both homeobox domains but lacking the C-terminal transactivation domain, closely resembling DUX4-s; it also carries a polyadenylation signal and shows 85.3% identity to DUX4FL.
- *Locus 2* predicts two alternative polypeptides depending on the reading frame used, but neither contains recognizable functional domains.

Taken together, these loci expand the potential repertoire of DUX4-related open reading frames beyond 4q/10q, ranging from near-identical paralogs to truncated isoforms reminiscent of DUX4c. Additionally, two paralogs (DUXL16 and DUXL18) were annotated at 10q11, and four (DUXL16–19) on Yq11, though their transcriptional and coding potential remain uncharacterized.

Strikingly, most D4Z4-like elements resolved by the T2T-CHM13 analysis display extremely high sequence identity (85.5–96.7%) with the canonical 4q35 and 10q26 arrays (Table 1), highlighting the challenge of designing locus-specific assays and the risk of conflating signals from paralogous loci.

### Assessing primer specificity when testing repetitive regions

Analysis presented in Table 1 raises critical concerns regarding the specificity of primers, probes, and antisense oligonucleotides (siRNA/shRNA) used in FSHD research ^11,38–41^: are these truly locus-specific, or could they also detect paralogous sequences elsewhere in the genome? This consideration extends beyond diagnostic and analytical assays to therapeutic applications, since several studies have explored siRNA-, shRNA- and ASO-based silencing of DUX4 and its downstream targets [9, 32]. Ensuring target specificity across D4Z4-like loci is therefore essential for the safe and effective development of RNA-directed therapies.

Transcriptional assays for DUX4FL typically rely on RT-PCR, qRT-PCR, or amplicon-based RNA sequencing. These often employ primers targeting the 3′ end of the transcript, particularly within exons 2/3 or the adjacent pLAM sequence (Fig. 1), in order to detect the PAS unique to the 4qA permissive allele. While this strategy is conceptually valid, the highly repetitive nature of the D4Z4 array complicates its applicability and interpretation. All internal repeats contain nearly identical copies of exon 1 and segments overlapping exon 2, and additional D4Z4-like elements are distributed across other chromosomes, notably 10q26. As a result, primers designed within exon 1 or exon 2 may anneal to multiple internal repeats or unrelated loci, generating amplicons that predominantly reflect non-coding transcripts. Even 3′-end-targeting primers can yield background signals due to alternative splicing or non-canonical PAS usage.

**Fig. 3:**
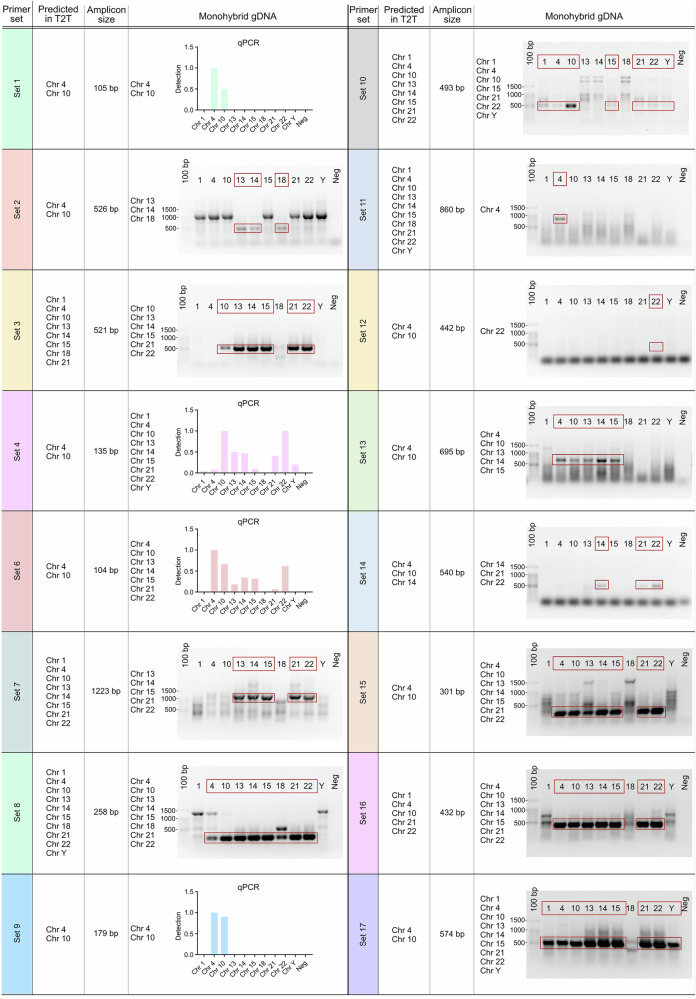
Primer tests using CHO monohybrid genomic DNA. Primer sets commonly used to amplify DUX4 (sets 1–4, 6–8) and DBE-T (sets 10–17) (see Supplementary Table. 1) were tested on genomic DNA from CHO monohybrids carrying chromosomes with D4Z4-like sequences (chr1, 3, 4 (4q35), 9, 10 (10q26), 13, 14, 15, 18, 21, 22, Y). Primer sets generating amplicons <200 bp (sets 1, 4, 6) were analyzed by qPCR, while those >200 bp (sets 2, 3, 7, 10–17) were analyzed by semi-quantitative PCR. For each primer set, the predicted target(s) identified by in silico PCR on T2T-CHM13 and the expected amplicon size are indicated. The expected PCR product is indicated by the red arrow, and chromosomes that tested positive are highlighted by red rectangles on gel photographs and listed alongside the data panels.

To systematically evaluate this issue, we tested primer pairs commonly used for DUX4FL detection, both in silico (hg38 vs. T2T-CHM13 references) and experimentally on genomic DNA from monochromosomal hybrids. Results are summarized in Supplementary Table 1 and Fig. 3, which show the amplicons produced by various primer sets using qPCR or semi-quantitative PCR, including those reported in previous studies [25, 27, 33–37].

Key findings include:

- Exon 1-targeting primers (sets 1 and 6): amplify all D4Z4 units on chromosomes 4 and 10, reflecting global array activity but lacking allele specificity.
- Exon 3-targeting primers: show divergent behaviors. Set 2 preferentially amplifies only the last repeat on 4q and 10q, enriching for terminal transcripts. By contrast, set 3 generates amplicons from additional loci across at least six other chromosomes. Set 8, whose reverse primer spans the exon 2/3 junction, is predicted to anneal to >120 sites across 10 different chromosomes in T2T-CHM13.
- pLAM-anchored assays (e.g., set 4): are comparatively more specific, since the PAS/poly(A) stretch is unique to the terminal repeat of 4qA alleles.
- Promoter- and DBE-targeting primers (sets 7, 9, 17): amplify all units on 4q/10q, consistent with the conservation of the DBE region, but set 7 also produces products from six additional chromosomes.

In summary, no primer set currently available ensures absolute allele- or locus-specific detection of pathogenic DUX4FL transcripts. Therefore, reliable attribution of transcript origin will require locus-resolved sequencing approaches, such as long-read RNA-seq or targeted capture of full-length transcripts. In addition, it should be noted that the detection of full-length DUX4 transcripts typically requires a nested PCR amplification step due to their extremely low and stochastic expression levels [6, 14, 38, 39]. While this approach increases sensitivity, it also enhances the risk of artefactual amplification in repetitive contexts, further underscoring the need for careful assay validation.

### Detection of the DBE-T transcript

The DBE-T long non-coding RNA, proposed to act as a chromatin modulator upstream of DUX4 activation, has been considered an indirect molecular marker of epigenetic dysregulation in FSHD. Its origin was mapped to a region immediately centromeric to the D4Z4 array [25].

As shown in Supplementary Table 1 and Fig. 3, we performed the same primer evaluation for DBE-T. Two primer sets uniquely target the Non-D4Z4 Element (NDE), located just proximal to the 4q35 array. In contrast, most of the other primers described in the literature anneal within internal D4Z4 repeats. Alignment to T2T-CHM13 reveals that these annealing sites are virtually identical across all units on chromosomes 4q and 10q, making discrimination of transcript origin impossible.

This redundancy imposes severe limitations: amplification from internal repeats cannot distinguish RNAs derived from 4q35, 10q26, or other paralogous loci. Consequently, many reported measurements of DBE-T risk conflating signals from multiple sources, introducing substantial ambiguity into its functional interpretation.

For reliable detection, DBE-T assays should be anchored to unique flanking regions (such as the NDE) or, preferably, employ long-read transcriptome sequencing to resolve repeat-derived RNAs at locus-specific resolution. Without such methodological precision, DBE-T quantification remains prone to misinterpretation, especially in studies linking its expression to disease severity or penetrance.

Focus Box 1 **Compelling question:**

***Are non-4q/10q D4Z4-like loci transcriptionally active?***

- *Evidence for transcriptional competence:* Early studies demonstrated that *internal* D4Z4 monomers can be transcribed in both sense and antisense orientations [22, 23] suggesting that any repeat unit could, in principle, serve as a promoter.
- *A more complex repeat landscape:* The T2T-CHM13 reference reveals dozens of near-identical D4Z4-like units outside 4q/10q, often embedded in subtelomeric or pericentromeric regions previously inaccessible to short-read mapping.
- *Detection caveats:* With few exceptions, widely used primers also amplify other D4Z4-like loci, so signals attributed to DBE-T or DUX4-related RNAs may in fact represent a mixture of transcripts from multiple chromosomes (Fig. 3). These RNAs can be detected irrespective of polyadenylation status, as shown by exon-1 assays that capture transcription from nearly every repeat.
- *Functional uncertainty:* No non-4q/10q locus has yet been conclusively shown to generate stable, polyadenylated DUX4FL transcripts. However, low-level or context-specific transcription from these repeats cannot be excluded.
- *Future directions*: Locus-resolved, long-read transcriptomics will be required to determine whether cryptic D4Z4-like loci contribute to the repeat-derived RNA pool in FSHD, and whether such transcription has regulatory or pathogenic consequences

## Discussion

### Clinical variability and the limits of a locus-centric model

FSHD exemplifies the difficulty of reconciling genetic architecture with clinical expression. While contraction of the 4q35 array below eight units on a permissive 4qA allele remains the strongest FSHD diagnostic marker [3], reduced arrays are relatively common in the general population ( ~ 3%) [40] and even more frequent in haplotype-resolved pangenomes (4,6%) [18, 20]. Thus, many carriers remain asymptomatic or present non-FSHD myopathic phenotypes, whereas some symptomatic individuals present with borderline or normal repeat sizes [41–43]. These observations underscore that the size of D4Z4 array alone cannot explain disease penetrance or progression, and that additional modifiers must be involved.

### Limitations of historical approaches

Several widely used strategies were developed under the assumption that D4Z4 repeats were restricted to 4q and 10q. CHO monohybrids carrying human chromosome 4q inserts provided early evidence for repeat-mediated silencing and array contraction. However, these models lack the native nuclear environment, are prone to drug-selection artifacts, and cannot recapitulate transcriptional features [44]. Likewise, SNP-based attribution of PCR amplicons has proven unreliable: the extreme sequence identity among monomers and the incompleteness of earlier reference genomes undermined locus assignment [14, 22, 25, 38]. A locus-centric view of FSHD was perpetuated by reliance on these incomplete models and strategies [3, 33]. Robust attribution now requires long-read, haplotype-resolved sequencing anchored to unique junctions and benchmarked against complete references such as T2T-CHM13 and emerging pangenomes.

### From a locus-centric to a repeat-aware framework

The completeness of the T2T-CHM13 reference [17] compels a shift from a 4q35-centric to a genome-wide, repeat-aware perspective. D4Z4-like units are widespread, highly similar in sequence, and frequently located in regions previously inaccessible to short-read methods. Under these conditions, assays designed within internal monomers or conserved promoter regions cannot guarantee locus specificity (see Supplementary Table 1 and Fig. 3). Sequence identity is the rule, not the exception, and results generated without locus-resolved approaches risk conflating signals from multiple chromosomes.

### Inter-individual variability and the need for de novo assemblies

T2T-CHM13 represents one single haploid reference genome and therefore cannot capture the extensive inter-individual variability of subtelomeric and pericentromeric repeats [45]. T2T-like assemblies, made available through large-scale initiatives such as the Human Pangenome Project, have already demonstrate copy number polymorphisms and structural rearrangements in these domains [18, 46]. Such variability is directly relevant to FSHD, as it alters the architecture and methylation context of D4Z4-like loci. In addition, comparative optical mapping and subtelomeric analyses across multiple populations [47] have revealed striking inter-individual variability in repeat organization and copy number, including at 4q, 10q, and acrocentric chromosomes. However, current optical mapping approaches (Bionano Genomics, 2023), remain limited by their reliance on *hg38*-based pipelines [48] which do not fully resolve highly repetitive loci such as D4Z4. These findings reinforce that the T2T reference likely represents only one of many possible D4Z4 configurations, and that population-level diversity may influence FSHD penetrance. Ultimately, haplotype-resolved, long-read assemblies for individual patients will be needed to reconstruct the true landscape of D4Z4 arrays and evaluate their contribution to clinical heterogeneity [49, 50].

### Consequences for diagnostics and biomarkers

For molecular diagnosis, locus attribution remains the critical bottleneck. The D4Z4 arrays at 4q35 and 10q26 are flanked by distinct proximal simple sequence length polymorphisms (SSLPs) that have been associated with the *qA* and *qB* alleles [51]. The *qA* configuration, in linkage with the non-canonical *ATTAAA* polyadenylation signal within the pLAM region, represents the genetic background historically associated with the stabilization of full-length *DUX4* transcripts in FSHD. Analysis of the T2T-CHM13 reference confirms that comparable SSLP structures are not found at other D4Z4-like loci. While this may contribute to the unique transcriptional competence of the 4q35 array, it does not necessarily imply that other repeat loci are transcriptionally inert.

Consistently with this view, our T2T-CHM13 survey (Table 1), together with pangenomic reconstructions [20], shows that non-canonical polyadenylation motifs, historically regarded as exclusive features of the 4qA permissive allele, also occur at selected non-4q D4Z4-like loci in specific haplotypes. Although these configurations lack complete pLAM cassettes and have not been shown to generate stable DUX4FL transcripts, their presence highlights that polyadenylation-competent architectures extend beyond chromosome 4 and may contribute to transcriptional diversity within the broader D4Z4 repeat family.

Instead, it highlights how differences in local sequence architecture and chromatin context can shape distinct transcriptional and/or translational outcomes.

Southern blotting and PFGE are robust because they assess the 4q35 haplotype in a locus-specific manner, but newer PCR-based or methylation assays are vulnerable to off-target effects. Transcript-based readouts suffer from similar limitations: exon 1 or promoter primers capture global array activity, while even pLAM-based designs require careful validation. Nested PCR, although sensitive, readily produces artefacts in a context where DUX4 expression is rare and stochastic [6, 14, 38, 39]. Current biomarkers also have shortcomings. The DUX4-induced gene expression signature, though reproducible, is sporadic and does not consistently correlate with clinical severity. DNA methylation-long considered a hallmark of D4Z4 repression-has been widely used, but bisulfite- and restriction-based assays target CpG-rich segments that are nearly identical across loci. For example, the commonly used “4qA-long/short” primers co-amplify non-4q repeats. Alignment to T2T-CHM13 shows that many reads map not only to 4q/10q but also to chromosomes 13, 15, or 21. These off-target arrays are consistently hypermethylated, while bona fide 4q signals show broad inter-sample variability. This explains why methylation estimates differ depending on whether GRCh38 or T2T is used [8, 20]. Because bisulfite conversion collapses sequence variation and the converted T2T-CHM13 genome shows >99% identity across all D4Z4-like repeats, most published methylation datasets necessarily reflect a composite signal from multiple paralogous loci. Consequently, the origin of ‘D4Z4 methylation’ in public data cannot be assumed to be 4qA-specific unless supported by haplotype-resolved long-read analyses. In this context, and in light of the multi-locus nature of bisulfite-derived signals, methylation cannot be used as a stand-alone criterion for diagnosis or clinical decision-making (this aspect is critical for prenatal diagnosis); it should instead be interpreted as a secondary parameter, integrated within a fully locus-resolved genetic framework. Moving forward, long-read amplicon sequencing with native methylation calling and unit-level resolution provides a robust alternative, capable of resolving true locus-specific methylation profiles. A clinically useful biomarker framework will need to integrate: (i) locus-resolved genotyping, (ii) unit-level methylation profiling, and (iii) reliable transcriptional or epigenetic markers. Equally important, clinically anchored resources such as patient registries and carefully stratified cohorts [52] provide the essential link between molecular biomarkers and disease variability.

### Beyond DUX4: a broader repeat-linked RNA program

While under the classical model full-length DUX4 is considered the sole pathogenic transcript, other repeat-linked RNAs are increasingly implicated in FSHD pathogenesis. DBE-T, transcribed proximal to the D4Z4 array [25], illustrates the technical challenges of discriminating transcript origin in a repetitive context. More recently, FRG2A has emerged as a paradigm of how D4Z4 derepression unleashes non-coding RNAs with architectural and translational functions [27]. FRG2A localizes to the nucleolus, forms subnuclear condensates, and interferes with ribosomal RNA synthesis, thereby impacting muscle protein translation. Importantly, FRG2A belongs to a family of 14 paralogs distributed across the genome, again raising the issue of paralog ambiguity [28]. These findings emphasize that derepression at 4q35 has consequences extending beyond protein-coding toxicity, reshaping nuclear organization and translational capacity. Beyond the canonical DUX4 paradigm, the multiplicity of transcriptionally competent D4Z4-like repeats and their associated non-coding RNAs may also contribute to the striking variability of clinical expression in FSHD. Variable expression or processing of *FRG2A* and other repeat-derived transcripts among individuals, even within the same family, might modulate nucleolar homeostasis, ribosome biogenesis, and translational output. Such inter-individual differences may influence the threshold for nucleolar stress and protein synthesis defects, providing a plausible mechanistic basis for the heterogeneous disease penetrance observed in patients carrying similar genetic lesions. Together, these observations reinforce the notion that FSHD arises from a multilayered regulatory network involving both coding and non-coding repeat-derived transcripts.

### Methodological shift and future directions

**Fig. 4:**
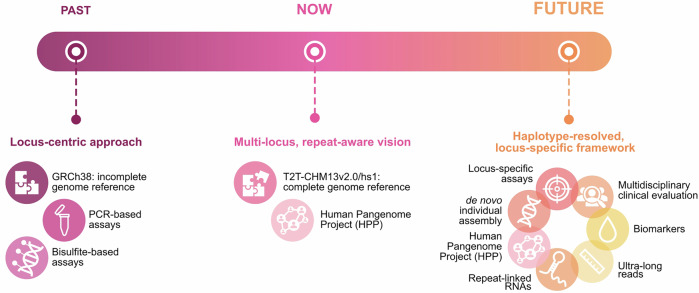
Timeline of FSHD research tools and knowledge. Over time, FSHD research has progressed from a limited, locus-centric view, constrained by few tools, to a more comprehensive understanding enabled by the T2T-CHM13v2.0/hs1 genome reference and the Human Pangenomes Project (HPP), which revealed numerous D4Z4-like sequences and a more complex genomic landscape. Today, we have unprecedented tools to study repetitive DNA at high resolution, integrating genetic, epigenetic, and transcriptomic data, including repeat-derived lncRNAs. It is crucial that future research fully leverages these tools, through de novo genome assembly, ultra-long read sequencing for haplotype-resolved analysis, patient-specific studies, and biomarker discovery, combining these approaches with accumulated knowledge to enhance diagnosis, patient stratification, and overall understanding of FSHD.

As illustrated in Fig. 4, the field has progressed from a locus-centric approach, based on incomplete references (GRCh38), PCR, and bisulfite assays, to a pan-locus, repeat-aware vision enabled by the complete T2T-CHM13 genome and pangenomic frameworks [17]. The next step will be the adoption of a haplotype-resolved, locus-specific approach, integrating de novo assemblies, ultra-long reads, locus-resolved assays, and repeat-linked RNA studies with multidisciplinary clinical evaluation. These insights converge on a clear conclusion: specificity is not optional when interrogating repetitive genomic regions. Locus-resolved methods are required at every level (genotype, CpG methylation, and transcriptome), anchored to unique junctions and validated through systematic in silico analyses.

In the context of current clinical care, the recently published consensus guideline on the genetic diagnostics of FSHD [53] provides a minimal framework for molecular confirmation of FSHD1 and FSHD2, by combining Southern blot/PFGE, allele-specific testing and methylation analysis. Current optical mapping approaches [54] allow high-confidence array sizing and haplotype resolution, yet they lack methylation information and do not detect structural variants in standard workflows, which only emerge upon manual de novo assembly. In contrast, long-read sequencing [55] now enables unit-level resolution of D4Z4 arrays, accurate repeat quantification, native methylation profiling and direct detection of structural variation. These innovations, illustrated in our revised schematic (Fig. 4), can enhance locus specificity, detect complex rearrangements and improve trial-readiness. Lastly, the concept of repeat-derived RNA signatures [28], as explored in this study, introduces a new biomarker dimension, which may enable personalised stratification of patients beyond the conventional genotype. Together, these layers form a future diagnostic–therapeutic continuum that holds promise for improved patient management, prognostication and tailored therapy.

Focus Box 2 **Practical recommendations**

- *Design principle:* Anchor primers/probes to unique flanking junctions or haplotype-specific polymorphisms; avoid internal D4Z4 sequences for locus attribution.
- *Technology choice*: Prefer long-read (ONT/PacBio) for both genotyping and transcriptomics; capture full-length isoforms and methylation in the same molecules when possible.
- *Mapping strategy:* Use repeat-aware aligners, report multi-mapping, and provide paralog/locus disambiguation criteria a priori.
- *Controls and models*: Include multi-locus controls (4q, 10q, selected non-4q/10q sites) and prioritize human isogenic systems over CHO hybrids.
- Reporting standards: Declare off-target analyses, primer in silico screens (vs. T2T-CHM13/pangenome), and criteria for calling DUX4-FL vs non-canonical products.

## Supplementary information

Supplemental Table 1

Supplemental Figure 1

## Data Availability

Correspondence and requests for materials should be addressed to Prof. Rossella Tupler (rossella.tupler@unimore.it). **Supplemental data:** Supplementary Data are available at EJHG online. They include Supplementary Table S1, listing all primer sets used and analyzed in this study, and Supplementary Fig. S1, illustrating the structure, coding potential, and domain conservation across D4Z4-related loci.
