# Supplementary material for: Rethinking genomics of facioscapulohumeral muscular dystrophy in the telomere-to-telomere era: pitfalls in the hidden landscape of D4Z4 repeats
Source: Eur J Hum Genet. 2026 Jan 14;34(3):357–67. doi: 10.1038/s41431-025-02000-x (PMC12963445; doi:10.1038/s41431-025-02000-x)
Supplement: Supplementary file 2 — Supplemental Figure 1 [file 41431_2025_2000_MOESM2_ESM.pdf]

A Complete (DBE-Ex1-Ex2-Ex3) and polyA containing D4Z4 elements

Chr	start	end	length	DBE-L	Exon1	Exon2	Exon3	PolyA signal	Putative Protein aa lenght	DUX4 like protein	ID
**chr4**	**193540105**	**193543625**	**3521**	✓	✓	✓	✓	✓	**485**	✓	
chr14	909434	912243	2810	✓	✓	✓	✓	-	167	-	
chr15	1030969	1033779	2811	✓	✓	✓	✓	-	167	-	
chr21	1050435	1053245	2811	✓	✓	✓	✓	-	364	-	
chr22	2327254	2330063	2810	✓	✓	✓	✓	-	130	-	
chr14	5370281	5373096	2816	✓	✓	✓	✓	-	240	-	
**chr10**	**134723013**	**134726548**	**3536**	✓	✓	✓	✓	-	**635**	✓	1
chr14	902466	905267	2802	✓	✓	✓	✓	-	230	-	
chr15	1024069	1026870	2802	✓	✓	✓	✓	-	230	-	
chr21	1043469	1046268	2800	✓	✓	✓	✓	-	230	-	
chr22	2320286	2323087	2802	✓	✓	✓	✓	-	230	-	
**chr1**	**128105722**	**128109167**	**3446**	✓	✓	✓	✓	-	**460**	✓	2
chr14	5400914	5403731	2818	✓	✓	✓	✓	-	236	-	
chr18	265409	266332	924	-	-	✓	✓	-	51	-	
chr21	6152912	6155730	2819	✓	✓	✓	✓	-	204	-	
chr22	1894840	1897663	2824	✓	✓	✓	✓	-	123	-	
chr22	6124174	6126992	2819	-	✓	✓	✓	-	207	-	
chr22	6235043	6237276	2234	✓	✓	✓	✓	-	84	-	
chr22	9489962	9492116	2155		✓	✓	✓	-	189	-	
chr22	9605546	9608371	2826	✓	✓	✓	✓	-	148	-	
chr14	5204889	5207707	2819	✓	✓	-	✓	-	149	-	
chr1	128153481	128156299	2819	✓	✓	-	✓	-	132	-	
chr1	128397121	128399942	2822	✓	✓	-	✓	-	174	-	
chr21	6145119	6147937	2819	✓	✓	-	✓	-	141	-	
chr21	6279265	6282084	2820	✓	✓	-	✓	-	231	-	
chr22	9477947	9480785	2839	✓	✓	-	✓	-	148	-	
chr22	9633040	9635860	2821	✓	✓	-	✓	-	203	-	
chr22	9639337	9642155	2819	✓	✓	-	✓	-	146	-	
chr22	6046725	6049542	2818	✓	✓	✓	-	✓	375	-	
chr14	4123281	4126102	2822	✓	✓	✓	-	✓	179	-	
chr14	4986889	4989300	2412	✓	✓	✓	-	✓	319	-	
chr22	9427115	9429913	2799	✓	✓	✓	-	✓	300	-	
**chr14**	**4161274**	**4164085**	**2812**	✓	✓	**-**	**-**	✓	**207**	✓	3
**chr14**	**4236370**	**4239197**	**2828**	✓	✓	**-**	**-**	✓	**379**	✓	4
**chr22**	**2383204**	**2386023**	**2820**	✓	✓	**-**	**-**	✓	**185**	✓	5
**chr22**	**6063372**	**6066189**	**2818**	✓	✓	**-**	**-**	✓	**232**	✓	6
chr22	6211604	6213214	1611	-	-	-	-	✓	116	-	

B. DUX4FL functional domains

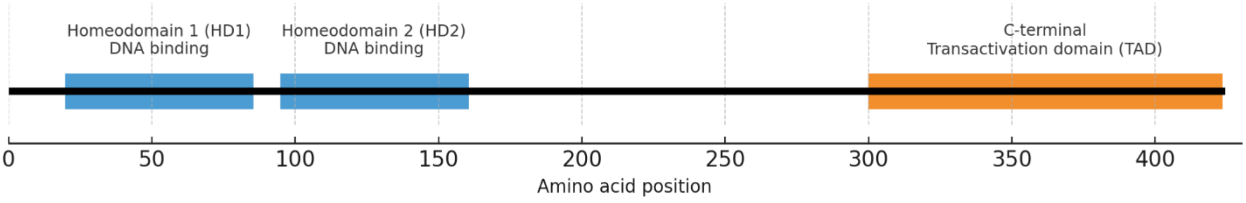

C. Functional domains of DUX4-like proteins

		DBE-L	Exon1	Exon2	Exon3	PolyA signal	aa lenght	HD1	HD2	TAD
1	chr10	✓	✓	✓	✓	-	422	✓	✓	✓
2	chr1	✓	✓	✓	✓	-	460	✓	✓	✓
3	chr14_1	✓	✓	-	-	✓	207	✓	-	-
4	chr14_2	✓	✓	-	-	✓	379	✓	✓	✓
5	chr22_1	✓	✓	-	-	✓	158	✓	✓	-
6.1	chr22_2_Frame1	✓	✓	-	-	✓	129	-	-	-
6.2	chr22_2_Frame3	✓	✓	-	-	✓	231	-	-	-
Ref.	chr4_last/DUX4FL	✓	✓	✓	✓	✓	424	✓	✓	✓
Ref.	chr4_DUX4c	✓	✓	✓	✓	-	374	✓	✓	-
Ref.	chr4_DUX4sh	-	✓	-	-	-	160	✓	✓	-

D. Percentage identity among DUX4-like proteins

		Percent Identity Matrix - created by Clustal 2.1									
		1	2	3	4	5	6.1	6.2	Ref.	Ref.	Ref.
1	chr10	100.00	80.57	74.88	79.95	82.28	85.27	78.02	100.00	94.65	99.38
2	chr1	80.57	100.00	70.05	76.52	72.15	73.64	72.41	80.57	80.75	83.75
3	chr14_1	74.88	70.05	100.00	65.70	69.62	75.19	35.00	74.88	74.88	81.25
4	chr14_2	79.95	76.52	65.70	100.00	74.68	73.64	68.23	79.95	77.27	83.75
5	chr22_1	82.28	72.15	69.62	74.68	100.00	73.64	-	82.28	82.28	82.28
6.1	chr22_2_Frame1	85.27	73.64	75.19	73.64	73.64	100.00	-	85.27	85.27	85.27
6.2	chr22_2_Frame3	78.02	72.41	35.00	68.23	-	-	100.00	78.02	70.05	-
Ref.	chr4_last/DUX4FL	100.00	80.57	74.88	79.95	82.28	85.27	78.02	100.00	94.65	99.38
Ref.	chr4_DUX4c	94.65	80.75	74.88	77.27	82.28	85.27	70.05	94.65	100.00	99.38
Ref.	chr4_DUX4sh	99.38	83.75	81.25	83.75	82.28	85.27	-	99.38	99.38	100.00

E. Multalin of ID1-6 sequences vs DUX4FL(chr4_last), DUXc and DUX4sh sequences

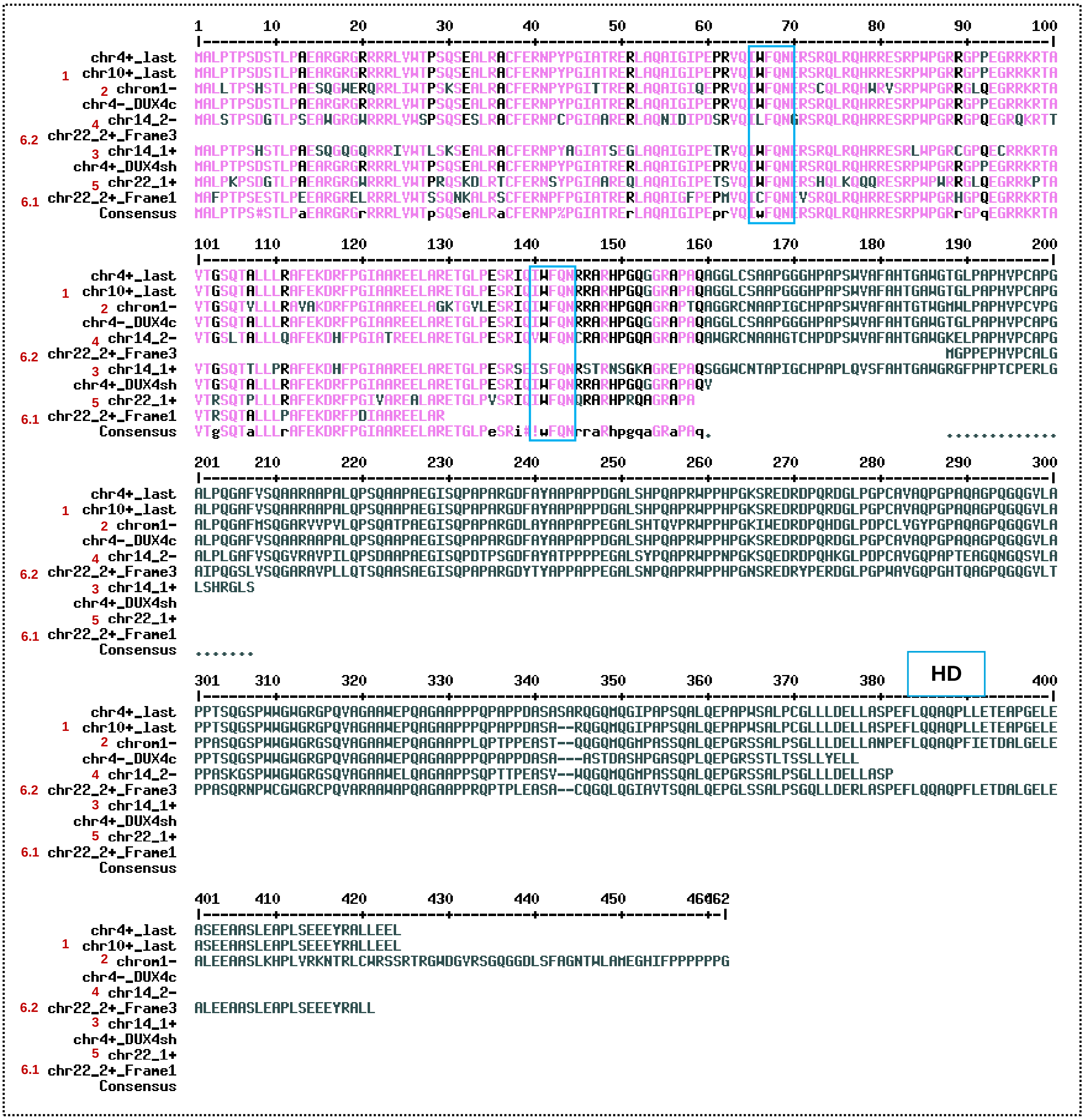

F. Multalin of ID1,2,4 vs DUX4FL(chr4_last) and DUXc sequences

```
               1        10        20        30        40        50        60        70        80        90        100
               |--------+---------+---------+---------+---------+---------+---------+---------+---------+---------|
   chr4+_last  MALPTPSDSTLPAEARGRGRRRRLVWTPSQSEALRACFERNPYPGIATRERLAQAIGIPEPRVQIWFQNERSRQLRQHRRESRPWPGRRGPPEGRRKRTA
 1 chr10+_last MALPTPSDSTLPAEARGRGRRRRLVWTPSQSEALRACFERNPYPGIATRERLAQAIGIPEPRVQIWFQNERSRQLRQHRRESRPWPGRRGPPEGRRKRTA
    2 chrom1-  MALLTPSHSTLPAESQGWERQRRLIWTPSKSEALRACFERNPYPGITTRERLAQAIGIQEPRVQIWFQNERSCQLRQHWRVSRPWPGRRGLQEGRRKRTA
  chr4-_DUX4c  MALPTPSDSTLPAEARGRGRRRRLVWTPSQSEALRACFERNPYPGIATRERLAQAIGIPEPRVQIWFQNERSRQLRQHRRESRPWPGRRGPPEGRRKRTA
   4 chr14_2-  MALSTPSDGTLPSEAWGRGWRRRLVWSPSQSESLRACFERNPCPGIAARERLAQNIDIPDSRVQILFQNGRSRQLRQHRRESRPWPGRRGPQEGRQKRTT
    Consensus  MALpTPSDSTLPAEArGRGRRRRLVWTPSQSEALRACFERNPYPGIATRERLAQAIGIPEPRVQIWFQNERSRQLRQHRRESRPWPGRRGPqEGRRKRTA

               101      110       120       130       140       150       160       170       180       190       200
               |--------+---------+---------+---------+---------+---------+---------+---------+---------+---------|
   chr4+_last  VTGSQTALLLRAFEKDRFPGIAAREELARETGLPESRIQIWFQNRRARHPGQGGRAPAQAGGLCSAAPGGGHPAPSWVAFAHTGAWGTGLPAPHVPCAPG
 1 chr10+_last VTGSQTALLLRAFEKDRFPGIAAREELARETGLPESRIQIWFQNRRARHPGQGGRAPAQAGGLCSAAPGGGHPAPSWVAFAHTGAWGTGLPAPHVPCAPG
    2 chrom1-  VTGSQTVLLLRAVAKDRFPGIAAREELAGKTGVLESRIQIWFQNRRARHPGQAGRAPTQAGGRCNAAPIGCHPAPSWVAFAHTGTWGMWLPAPHVPCVPG
  chr4-_DUX4c  VTGSQTALLLRAFEKDRFPGIAAREELARETGLPESRIQIWFQNRRARHPGQGGRAPAQAGGLCSAAPGGGHPAPSWVAFAHTGAWGTGLPAPHVPCAPG
   4 chr14_2-  VTGSLTALLLQAFEKDHFPGIATREELARETGLPESRIQVWFQNCRARHPGQAGRAPAQAWGRCNAAHGTCHPDPSWVAFAHTGAWGKELPAPHVPCAPG
    Consensus  VTGSQTALLLRAFEKDRFPGIAAREELARETGLPESRIQIWFQNRRARHPGQaGRAPAQAGGrCnAAPGGcHPAPSWVAFAHTGAWGtgLPAPHVPCAPG

               201      210       220       230       240       250       260       270       280       290       300
               |--------+---------+---------+---------+---------+---------+---------+---------+---------+---------|
   chr4+_last  ALPQGAFVSQAARAAPALQPSQAAPAEGISQPAPARGDFAYAAPAPPDGALSHPQAPRWPPHPGKSREDRDPQRDGLPGPCAVAQPGPAQAGPQGQGVLA
 1 chr10+_last ALPQGAFVSQAARAAPALQPSQAAPAEGISQPAPARGDFAYAAPAPPDGALSHPQAPRWPPHPGKSREDRDPQRDGLPGPCAVAQPGPAQAGPQGQGVLA
    2 chrom1-  ALPQGAFMSQGARVVPVLQPSQATPAEGISQPAPARGDLAYAAPAPPEGALSHTQVPRWPPHPGKIWEDRDPQHDGLPDPCLVGYPGPAQAGPQGQGVLA
  chr4-_DUX4c  ALPQGAFVSQAARAAPALQPSQAAPAEGISQPAPARGDFAYAAPAPPDGALSHPQAPRWPPHPGKSREDRDPQRDGLPGPCAVAQPGPAQAGPQGQGVLA
   4 chr14_2-  ALPLGAFVSQGVRAVPILQPSDAAPAEGISQPDTPSGDFAYATPPPPEGALSYPQAPRWPPNPGKSQEDRDPQHKGLPDPCAVGQPAPTEAGQNGQSVLA
    Consensus  ALPQGAFVSQgARAvPaLQPSQAAPAEGISQPAPARGDFAYAAPAPP#GALSHPQAPRWPPHPGKSrEDRDPQhDGLPdPCAVgQPGPAQAGPQGQGVLA

               301      310       320       330       340       350       360       370       380       390       400
               |--------+---------+---------+---------+---------+---------+---------+---------+---------+---------|
   chr4+_last  PPTSQGSPWHGWGRGPQVAGAAWEPQAGAAPPPQPAPPDASASARQGQMQGIPAPSQALQEPAPWSALPCGLLLDELLASPEFLQQAQPLLETEAPGELE
 1 chr10+_last PPTSQGSPWHGWGRGPQVAGAAWEPQAGAAPPPQPAPPDASA--RQGQMQGIPAPSQALQEPAPWSALPCGLLLDELLASPEFLQQAQPLLETEAPGELE
    2 chrom1-  PPASQGSPWHGWGRGSQVAGAAWEPQAGAAPPLQPTPPEAST--QQGQMQGMPASSQALQEPGRSSALPSGLLLDELLANPEFLQQAQPFIETDALGELE
  chr4-_DUX4c  PPTSQGSPWHGWGRGPQVAGAAWEPQAGAAPPPQPAPPDASA---ASTDASHPGASQPLQEPGRSSTLTSSLLYELL
   4 chr14_2-  PPASKGSPWHGWGRGSQVAGAAWELQAGAAPPSQPTTPEASV--WQGQMQGMPASSQALQEPGRSSALPSGLLLDELLASP
    Consensus  PPaSQGSPWHGWGRGsQVAGAAWEPQAGAAPPpQPtPP#ASa  wQGQMQGmPAsSQALQEPGRSSALPSGLLLDELLAsP

               401      410       420       430       440       450      46062
               |--------+---------+---------+---------+---------+---------+-|
   chr4+_last  ASEEAASLEAPLSEEEYRALLEEL
 1 chr10+_last ASEEAASLEAPLSEEEYRALLEEL
    2 chrom1-  ALEEAASLKHPLVRKNTRLCWRSSRTRGWDGVRSGQGGDLSFAGNTWLAMEGHIFPPPPPPG
  chr4-_DUX4c
   4 chr14_2-
    Consensus
```

HD TAD

G. aa sequences of selected proteins

```
Reference>chr4+_last        424aa
MALPTPSDSTLPAEARGRGRRRRLVWTPSQSEALRACFERNPYPGIATRE
RLAQAIGIPEPRVQIWFQNERSRQLRQHRRESRPWPGRRGPPEGRRKRTA
VTGSQTALLLRAFEKDRFPGIAAREELARETGLPESRIQIWFQNRRARHP
GQGGRAPAQAGGLCSAAPGGGHPAPSWVAFAHTGAWGTGLPAPHVPCAPG
ALPQGAFVSQAARAAPALQPSQAAPAEGISQPAPARGDFAYAAPAPPDGA
LSHPQAPRWPPHPGKSREDRDPQRDGLPGPCAVAQPGPAQAGPQGQGVLA
PPTSQGSPWWGWGRGPQVAGAAWEPQAGAAPPPQPAPPDASASARQGQMQ
GIPAPSQALQEPAPWSALPCGLLLDELLASPEFLQQAQPLLETEAPGELE
ASEEAASLEAPLSEEEYRALLEEL
```

```
Reference>chr4+_DUX4sh        160 aa
MALPTPSDSTLPAEARGRGRRRRLVWTPSQSEALRACFERNPYPGIATRE
RLAQAIGIPEPRVQIWFQNERSRQLRQHRRESRPWPGRRGPPEGRRKRTA
VTGSQTALLLRAFEKDRFPGIAAREELARETGLPESRIQIWFQNRRARHP
GQGGRAPAQV----------------------------------------
--------------------------------------------------
--------------------------------------------------
--------------------------------------------------
--------------------------------------------------
--------------------------------------------------
------------
```

```
Reference>chr4-_DUX4c        374aa
MALPTPSDSTLPAEARGRGRRRRLVWTPSQSEALRACFERNPYPGIATRE
RLAQAIGIPEPRVQIWFQNERSRQLRQHRRESRPWPGRRGPPEGRRKRTA
VTGSQTALLLRAFEKDRFPGIAAREELARETGLPESRIQIWFQNRRARHP
GQGGRAPAQAGGLCSAAPGGGHPAPSWVAFAHTGAWGTGLPAPHVPCAPG
ALPQGAFVSQAARAAPALQPSQAAPAEGISQPAPARGDFAYAAPAPPDGA
LSHPQAPRWPPHPGKSREDRDPQRDGLPGPCAVAQPGPAQAGPQGQGVLA
PPTSQGSPWWGWGRGPQVAGAAWEPQAGAAPPPQPAPPDASA---ASTDA
SHPGASQPLQEPGRSSTLTSSLLYELL-----------------------
--------------------------------------------------
------------
```

```
1>chr10+_last        422aa
MALPTPSDSTLPAEARGRGRRRRLVWTPSQSEALRACFERNPYPGIATRE
RLAQAIGIPEPRVQIWFQNERSRQLRQHRRESRPWPGRRGPPEGRRKRTA
VTGSQTALLLRAFEKDRFPGIAAREELARETGLPESRIQIWFQNRRARHP
GQGGRAPAQAGGLCSAAPGGGHPAPSWVAFAHTGAWGTGLPAPHVPCAPG
ALPQGAFVSQAARAAPALQPSQAAPAEGISQPAPARGDFAYAAPAPPDGA
LSHPQAPRWPPHPGKSREDRDPQRDGLPGPCAVAQPGPAQAGPQGQGVLA
PPTSQGSPWWGWGRGPQVAGAAWEPQAGAAPPPQPAPPDASA--RQGQMQ
GIPAPSQALQEPAPWSALPCGLLLDELLASPEFLQQAQPLLETEAPGELE
ASEEAASLEAPLSEEEYRALLEEL
```

```
2>chrom1-        460aa
MALLTPSHSTLPAESQGWERQRRLIWTPSKSEALRACFERNPYPGITTRE
RLAQAIGIQEPRVQIWFQNERSCQLRQHWRVSRPWPGRRGLQEGRRKRTA
VTGSQTVLLLRAVAKDRFPGIAAREELAGKTGVLESRIQIWFQNRRARHP
GQAGRAPTQAGGRCNAAPIGCHPAPSWVAFAHTGTWGMWLPAPHVPCVPG
ALPQGAFMSQGARVVPVLQPSQATPAEGISQPAPARGDLAYAAPAPPEGA
LSHTQVPRWPPHPGKIWEDRDPQHDGLPDPCLVGYPGPAQAGPQGQGVLA
PPASQGSPWWGWGRGSQVAGAAWEPQAGAAPPLQPTPPEAST--QQGQMQ
GMPASSQALQEPGRSSALPSGLLLDELLANPEFLQQAQPFIETDALGELE
ALEEAASLKHPLVRKNTRLCWRSSRTRGWDGVRSGQGGDLSFAGNTWLAM
EGHIFPPPPPPG
```

```
3>chr14_1+       207aa
MALPTPSHSTLPAESQGQGQRRRIVWTLSKSEALRACFERNPYAGIATSE
GLAQAIGIPETRVQIWFQNERSRQLRQHRRESRLWPGRCGPQECRRKRTA
VTGSQTTLLPRAFEKDHFPGIAAREELARETGLPESRSEISFQNRSTRNS
```

```
GKAGREPAQSGGWCNTAPIGCHPAPLQVSFAHTGAWGRGFPHPTCPERLG
LSHRGLS-------------------------------------------
--------------------------------------------------
--------------------------------------------------
--------------------------------------------------
--------------------------------------------------
------------
```

4>chr14_2-   379aa

```
MALSTPSDGTLPSEAWGRGWRRRLVWSPSQSESLRACFERNPCPGIAARE
RLAQNIDIPDSRVQILFQNGRSRQLRQHRRESRPWPGRRGPQEGRQKRTT
VTGSLTALLLQAFEKDHFPGIATREELARETGLPESRIQVWFQNCRARHP
GQAGRAPAQAWGRCNAAHGTCHPDPSWVAFAHTGAWGKELPAPHVPCAPG
ALPLGAFVSQGVRAVPILQPSDAAPAEGISQPDTPSGDFAYATPPPPEGA
LSYPQAPRWPPNPGKSQEDRDPQHKGLPDPCAVGQPAPTEAGQNGQSVLA
PPASKGSPWWGWGRGSQVAGAAWELQAGAAPPSQPTTPEASV--WQGQMQ
GMPASSQALQEPGRSSALPSGLLLDELLASP-------------------
--------------------------------------------------
------------
```

5>chr22_1+   158 aa

```
MALPKPSDGTLPAEARGRGWRRRLVWTPRQSKDLRTCFERNSYPGIAARE
QLAQAIGIPETSVQIWFQNERSHQLKQQQRESRPWPWRRGLQEGRRKPTA
VTRSQTPLLLRAFEKDRFPGIVAREALARETGLPVSRIQIWFQNQRARHP
RQAGRAPA------------------------------------------
--------------------------------------------------
--------------------------------------------------
--------------------------------------------------
--------------------------------------------------
--------------------------------------------------
------------
```

6.1>chr22_2+_Frame1   129 aa

```
MAFPTPSESTLPEEARGRELRRRLVWTSSQNKALRSCFERNPFPGIATRE
RLAQAIGFPEPMVQICFQNEVSRQLRQHRRESRPWPGRHGPQEGRRKRTA
VTRSQTALLLPAFEKDRFPDIAAREELAR---------------------
--------------------------------------------------
--------------------------------------------------
--------------------------------------------------
--------------------------------------------------
--------------------------------------------------
--------------------------------------------------
------------
```

6.2>chr22_2+_Frame3   231aa

```
--------------------------------------------------
--------------------------------------------------
--------------------------------------------------
-------------------------------------MGPPEPHVPCALG
AIPQGSLVSQGARAVPLLQTSQAASAEGISQPAPARGDYTYAPPAPPEGA
LSNPQAPRWPPHPGNSREDRYPERDGLPGPWAVGQPGHTQAGPQGQGVLT
PPASQRNPWCGWGRCPQVARAAWAPQAGAAPPRQPTPLEASA--CQGQLQ
GIAVTSQALQEPGLSSALPSGQLLDERLASPEFLQQAQPFLETDALGELE
ALEEAASLEAPLSEEEYRALL-----------------------------
------------
```

Supplementary Figure S1. Structure, coding potential, and domain conservation across D4Z4-related loci.

A, **Annotation of D4Z4-like loci.** Table summarizing genomic coordinates, chromosomal location, and structural organization of D4Z4-related elements as canonical tandem units or isolated monomers. For each locus, the presence of DBE, Exon 1–3, and a polyadenylation signal is indicated together with predicted protein length. Two recurrent configurations emerge: (i) structurally complete repeats containing the promoter, Exon 1, Exon 2 and Exon 3, but lacking a polyadenylation signal, and (ii) repeats containing a polyA signal but incomplete downstream cassette (missing Exon 2+3 or Exon 3 alone). In addition to the well-characterized chromosome 10 locus, five additional loci (ID2–ID6) with the potential to encode DUX4-like proteins are identified. **B**, **DUX4FL functional organization.** Schematic representation of the DUX4 full-length protein showing the two N-terminal homeodomains (HD1 and HD2) and the C-terminal transactivation domain (TAD). **C**, **Domain conservation across DUX4-like proteins.** Predicted proteins encoded by loci ID1–ID6 are compared with reference proteins DUX4FL, DUX4c, and the short isoform DUX4short. The presence or absence of HD1, HD2, and TAD is indicated, revealing heterogeneity in domain architecture.
D, **Sequence identity matrix.** Pairwise percentage identity among DUX4-like proteins (IDs 1–6) and reference sequences (DUX4FL, DUX4c, DUX4short), generated using Clustal 2.1. While some loci retain >80% identity with DUX4-FL across homeodomains, truncated forms (e.g. ID3, ID5, ID6.1) display higher similarity to the short isoform DUX4-s, reflecting the absence of the C-terminal transactivation domain.
E, **Multalin alignment of complete ORFs.** Alignment of predicted full-length coding sequences (IDs 1, 2, 4, 6.2) with DUX4-FL, DUX4c, and DUX4short. Conserved motifs, including the hallmark IWFQN boxes in HD1 and HD2 and the TAD region, are highlighted, supporting their classification as bona fide DUX4-like proteins.
F, **Multalin alignment of incomplete sequences.** Alignment of truncated or partial ORFs (IDs 3, 5, 6.1), showing that although complete domain composition is lacking, partial homeodomain conservation is retained. **G**, **Reference amino acid sequences.** Complete amino acid sequences of selected DUX4-like proteins are reported alongside reference proteins. Functionally relevant regions are annotated: homeodomains (highlighted in blue) and the transactivation domain (highlighted in red).
